# A Resonant Pressure Microsensor with a Wide Pressure Measurement Range

**DOI:** 10.3390/mi12040382

**Published:** 2021-04-01

**Authors:** Chao Xiang, Yulan Lu, Chao Cheng, Junbo Wang, Deyong Chen, Jian Chen

**Affiliations:** 1 Aerospace Information Research Institute, Chinese Academy of Sciences, Beijing 100190, China; xiangchao115@mails.ucas.ac.cn (C.X.); luyulan15@mails.ucas.ac.cn (Y.L.); chengchao18@mails.ucas.ac.cn (C.C.); chenjian@mail.ie.ac.cn (J.C.); 2 School of Electronic, Electrical and Communication Engineering, University of Chinese Academy of Sciences, Beijing 100049, China

**Keywords:** resonant pressure microsensor, wide pressure measurement range, silicon islands, cylindrical vacuum cavity, quality factor

## Abstract

This paper presents a resonant pressure microsensor with a wide range of pressure measurements. The developed microsensor is mainly composed of a silicon-on-insulator (SOI) wafer to form pressure-sensing elements, and a silicon-on-glass (SOG) cap to form vacuum encapsulation. To realize a wide range of pressure measurements, silicon islands were deployed on the device layer of the SOI wafer to enhance equivalent stiffness and structural stability of the pressure-sensitive diaphragm. Moreover, a cylindrical vacuum cavity was deployed on the SOG cap with the purpose to decrease the stresses generated during the silicon-to-glass contact during pressure measurements. The fabrication processes mainly contained photolithography, deep reactive ion etching (DRIE), chemical mechanical planarization (CMP) and anodic bonding. According to the characterization experiments, the quality factors of the resonators were higher than 15,000 with pressure sensitivities of 0.51 Hz/kPa (resonator I), −1.75 Hz/kPa (resonator II) and temperature coefficients of frequency of 1.92 Hz/°C (resonator I), 1.98 Hz/°C (resonator II). Following temperature compensation, the fitting error of the microsensor was within the range of 0.006% FS and the measurement accuracy was as high as 0.017% FS in the pressure range of 200 ~ 7000 kPa and the temperature range of −40 °C to 80 °C.

## 1. Introduction

In the fields of industrial control, aerospace and deep-sea exploration, pressure sensors with a wide measurement range (over several atmospheric pressure) are widely used [1]. These pressure sensors mainly function with several typical working principles, such as piezoresistive sensing [2,3,4], piezoelectric sensing [5,6], capacitive sensing [7,8], fiber optical sensing [9,10] and resonant sensing [11,12,13,14,15,16,17]. Piezoresistive pressure sensors are widely used in many areas because of significant advantages, including high sensitivities, fast dynamic responses and easy miniaturizations. Nevertheless, piezoresistive pressure sensors are sensitive to temperature variations and thus suffer from limited resolutions [18]. Compared with piezoresistive pressure sensors, although piezoelectric pressure sensors are not sensitive to temperature variations, they cannot measure pressures without variations as a function of time [19]. As another type of pressure sensor which is not sensitive to temperature variations, a capacitive pressure sensor requires complex detection circuits [20]. Fiber optical pressure sensors are immune to electromagnetic interferences, and thus they can work in harsh environments. However, the fabrication of fiber optical pressure sensors is quite complex [21]. Compared to other kinds of pressure sensors, resonant pressure sensors feature high accuracies, high resolutions, quasi-digital outputs and high long-term stabilities [22,23,24,25,26].

J.C. Greenwood presented a silicon resonant sensor for measuring a wide pressure range up to 48.265 MPa in 1994 [27]. The resolution of this sensor was better than 100 ppm with the total errors better than 0.02% FS from −20 °C to 70 °C [28]. In 2009, P.K. Kinnell presented a Micro-Electro-Mechanical System (MEMS) resonant pressure sensor employing a flexible fabrication route, including direct silicon fusion bonding and deep reactive ion etching (DRIE) [29]. Theoretically, the sensor can measure 700 bar (70,000 kPa) in a hermetic package. Experimental results showed that the total errors of the sensor were less than 40 ppm over a temperature range of −54 °C to 125 °C and a pressure range of 0 to 200 kPa. Yiwen Jiang presented a micromachined resonant pressure sensor based on a fully symmetrical structure in 2012 [30]. Experimental results showed that the sensor had a pressure sensitivity of about 22.6 Hz/kPa, within the pressure range up to 550 kPa. In addition, H. Mitsuya introduced a resonant pressure sensor based on squeeze-film damping in a 2 µm driving/sensing gap of a silicon ring-shaped resonator in 2014 [31]. The reported data showed that the sensitivity was 1.8 Hz/kPa under a measurement range of 10 kPa to 1 MPa. Furthermore, our previous studies focused on several types of resonant pressure sensors with pressure measurement ranges over 500 kPa, such as Lu (2019) [32], Yan (2019) [33] and Xiang (2020) [34], with s maximal value of 2 MPa.

When the range of pressure measurements is expanded from 0~2 MPa to 0~7 MPa, the maximum deformation of the pressure-sensitive diaphragm is over several micrometers. Therefore, the microsensor works in a nonlinear state and the accuracy of the microsensor decreases. If the maximum stress on the pressure-sensitive diaphragm is higher than the breaking strength of silicon, the microsensor can be damaged permanently. Hence, the key points of expanding the pressure measurement range of the microsensor are reasonable design of the microsensor’s structures, regulating pressure sensitivities and increasing structural strength of the microsensor.

In order to address this issue, this study presented a resonant pressure microsensor based on a specific design to downregulate the pressure sensitivities, improve the structural strength of the microsensor and eventually further enlarge the pressure measurement range. The specific design optimized the shape of the vacuum cavity and the parameter of the resonant beams. By employing this specific design, the pressure measurement errors of this microsensor were less than 0.02% FS from 80 °C to −40 °C and over a pressure range of 200 to 7000 kPa. Moreover, compared with the resonant pressure sensors which were reported before, the package of this microsensor was easy to conduct. This paper (1) introduced the design of the microsensor, (2) estimated maximum stresses under high pressures and obtained frequency responses of the resonant microsensor based on theoretical calculations and numerical simulations, (3) realized the fabrication of the microsensor based on MEMS, (4) validated the microsensor by employing several experiments including open-loop and closed-loop tests.

## 2. Theoretical Analysis

### 2.1. Working Principle

**Figure 1:**
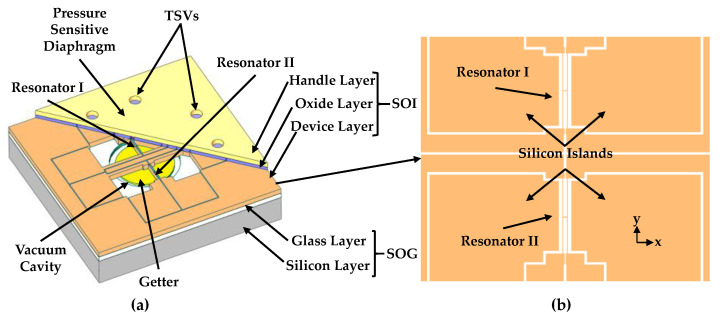
(**a**) Schematic of the resonant pressure sensor, including a silicon-on-insulator (SOI) layer and a silicon-on-glass (SOG) cap. In the SOI wafer, there is a pressure-sensitive diaphragm, eight TSVs in the handle layer and two H-shaped doubly clamped resonators in the device layer. In the device layer of the SOI, four silicon islands are hidden to show the vacuum cavity. The SOG cap with a cavity, evaporated with the getter material, was used to form vacuum packaging for the resonators and reduce the temperature sensitivity of the resonant pressure sensor; (**b**) detailed structure of sensing elements.

As shown in Figure 1a, the proposed resonant pressure sensor mainly consists of a silicon-on-insulator (SOI) layer as sensing elements and a silicon-on-glass (SOG) cap (including a glass layer and a silicon layer) with a cavity for vacuum packaging. The sensing elements include a pressure-sensitive diaphragm and a pair of resonators. To keep the two resonators working linearly in a wide pressure measurement range and increase the structural strength of the microsensor to bear larger pressures, the whole handle layer functions as a pressure-sensitive diaphragm for the purpose of increasing the thickness of the pressure-sensitive diaphragm. Besides, the device layer is used to form a pair of H-shaped doubly clamped resonators (including “resonator I” located in the central areas of the diaphragm and “resonator II” located in the edge areas of the diaphragm, respectively). Additionally, electronic connections are formed by eight through silicon vias (TSVs). The SOG cap with a cylindrical cavity could strongly reduce the stress between the SOI layer and the SOG layer during high-pressure measurements. Meanwhile, four silicon islands are designed in the device layer of the SOI wafer. The silicon islands can be regarded as a part of the pressure-sensitive diaphragm, which can increase the thickness of the pressure-sensitive diaphragm further (as shown in Figure 1b).

**Figure 2:**
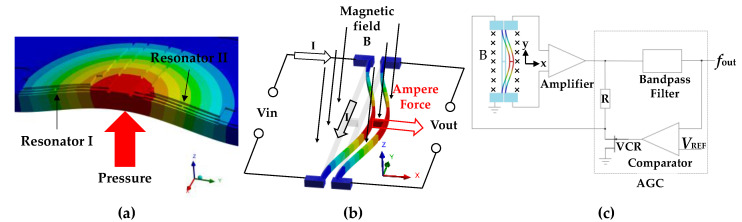
(**a**) The stress distribution on sensing elements under pressure; (**b**) the working principle of the resonator; (**c**) the working principle of the presented microsensor.

Pressure under measurement causes the deformation of the pressure-sensitive diaphragm, changing the axial stress of the resonators (see Figure 2a). Hence, the intrinsic resonant frequencies of the two resonators shift. By measuring these two resonant frequencies, the pressure can be calculated. Meanwhile, differential outputs of the two resonators’ frequencies can decrease the influence caused by temperature variations and increase the pressure sensitivities of the pressure measurements.

According to Figure 1, the resonators of the presented microsensor are two “H” beams. These two resonators vibrate laterally at the first-order modal (see Figure 2b).

The proposed microsensor was based on electro-magnetic excitation/electro-magnetic detection. Additionally, the detailed detection principle was as follows. Carrying an AC current, the resonator vibrated due to Ampère’s force, in a static magnetic field which is perpendicular to the pressure-sensitive diaphragm. The resulting vibration of the resonator produced a magnetic induction voltage, which was further processed with the help of amplifier and bandpass filters, for the extraction of the frequency signals (see Figure 2c). Then, the comparison between the magnitude of the output frequency signal and a constant voltage (V_REF_) was used to control the resistance of a voltage-controlled resistor (VCR). Therefore, the driving signal of the resonator can be controlled to keep outputting a signal with constant magnitude. This principle of the circuit is called automatic gain control (AGC). These two output frequencies of the microsensor are functions of pressure and temperature. After calibration, employing a polynomial fitting method based on differential outputs and a temperature sensor, the microsensor’s temperature compensation can be achieved in a wide pressure measurement range [34].

### 2.2. Optimal Design and Finite Element Analysis (FEA) Simulations

A wide pressure measurement range means the microsensor should work in a high-pressure environment. The microsensor presented in this paper was designed to work from 200 kPa to 7000 kPa. Therefore, the design must ensure that the microsensor can work stably under 7000 kPa. In order to analyze the status of the microsensor working at 7000 kPa, theoretic analysis was employed.

According to structural mechanics [35], the maximum stresses of the microsensor at the interface between silicon and glass should be

(1) $${\left(\sigma_{rr}\right)}_{\max}=\alpha \cdot \frac{A}{h^{2}}P,$$

where *α* is the parameter of the pressure-sensitive diaphragm shape as shown in Table 1, *P* is the outside pressure, *A* is area of the pressure-sensitive diaphragm, and *h* is the thickness of the pressure-sensitive diaphragm.

Equation (1) shows that the maximum stress on the microsensor mainly depended on the area and the shape of the pressure-sensitive diaphragm. Additionally, the stress on a circular pressure-sensitive diaphragm was smaller than the square counterpart of the same area.

To verify the design of the microsensor, FEA simulations based on ANSYS were employed to evaluate the maximum stress on the microsensors with two different pressure-sensitive diaphragms. The diameter of the circular pressure-sensitive diaphragm was equal to the side length of the square pressure-sensitive diaphragm.

**Figure 3:**
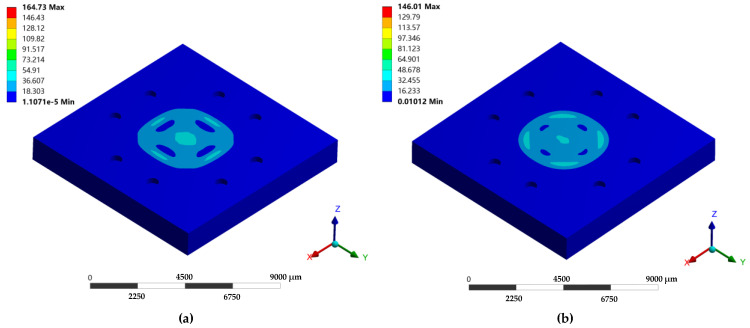
(**a**) Structural simulations under 7000 kPa: (**a**) the microsensor with square pressure-sensitive diaphragm (side length of 4.4 mm); (**b**) the microsensor with circular pressure-sensitive diaphragm (diameter of 4.4 mm).

Figure 3a,b proves that the maximum stress on the circular pressure-sensitive diaphragm was smaller than the stress on the square counterpart under 7000 kPa.

For the sake of choosing a suitable size of vacuum cavity to make the microsensor work well at 7 MPa, several chips with different vacuum cavity sizes were fabricated to conduct pressure loading experiments. The parameters of these chips are listed in Table 2. The shape of type A and B was a square, while the shape of type C and D was a circle.

Table 3 shows the experimental results with different sizes under several pressure points. When the diameter of the vacuum cavity was 3.6 mm, the maximum safe loading pressure can reach 40 MPa, which suggests the strength of the microsensor can meet the requirement. Therefore, the diameter of the presented microsensor’s vacuum cavity was set as 3.56 mm.

The dimensional information of the microsensor model in FEA simulations is listed in Table 4.

Several simulations were also conducted to calculate the intrinsic frequency shifts in response to pressure and temperature variations, relying on multi-models of steady-state thermal, static structural, and modal. In simulations, tetrahedral elements were used to mesh geometrical structures of the microsensor with 2,265,845 elements.

The materials used in simulations are listed in Table 5. In addition, a meshing size of 100 μm was used to mesh the entire body. Finally, the resonant frequencies were calculated as the temperature dropped from bonding temperature (350 °C) to an ambient working temperature range (−40~80 °C).

Resonator I was designed to measure the pressure change. Meanwhile, resonator II was designed to realize temperature compensation. In the static structure, pressures from 200 kPa to 7000 kPa were used as the loads. The generated stress distributions within the structures were then used as the loads for the modal analysis, and the outputs of the simulations were the intrinsic frequency shifts of the resonators in response to the pressure applied.

The initial conditions of temperature properties in simulations were set with a reference temperature of 350 °C, which is the anodic bonding temperature of the SOG cap and the SOI wafer. In fact, in order to facilitate the comparison with experimental results, a reference pressure of 100 kPa was introduced into the static structure. In the steady-state thermal simulations, temperatures from −40 to 80 °C were used as the loads and the temperature distributions of the whole structure were transferred to the static structure. The calculated stresses within the structures were then used as the loads for the modal analysis, and the outputs of the simulations were the intrinsic frequencies of the resonators in response to temperature variances.

**Figure 4:**
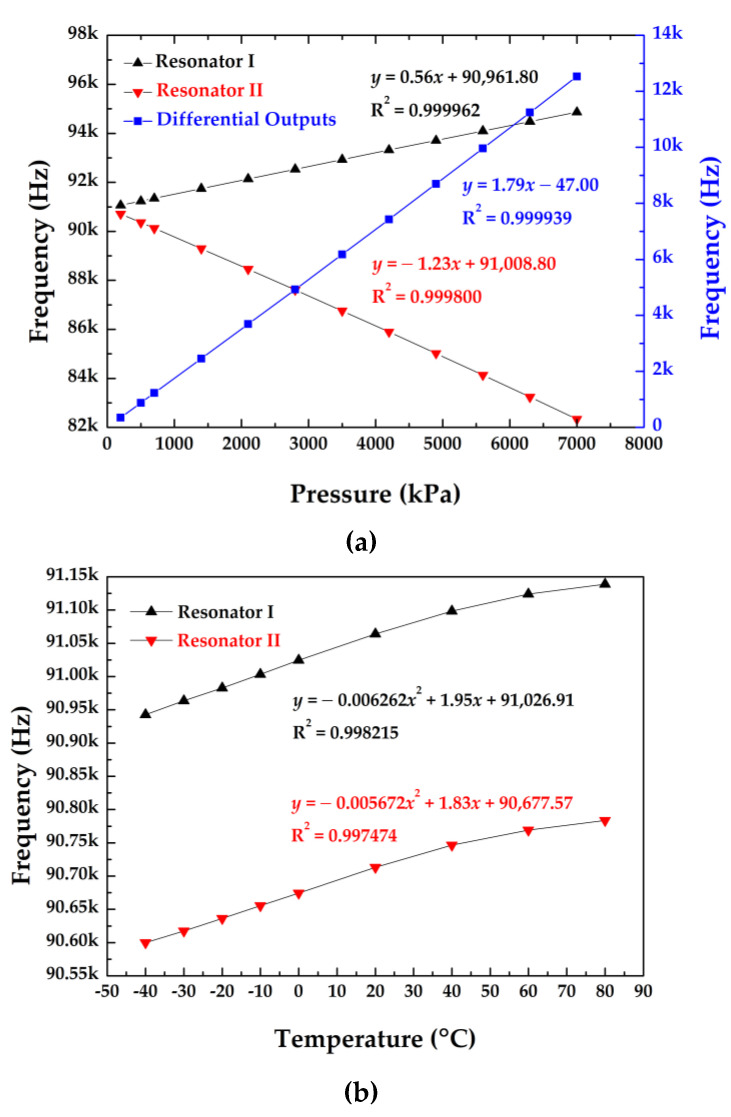
(**a**) The pressure–frequency curve of the developed sensor at 20 °C; (**b**) the temperature–frequency curve of the developed sensor under 100 kPa.

In addition, the intrinsic frequency shifts as a function of applied pressures and surrounding temperature variations are shown in Figure 4a,b. The pressure sensitivities of the two resonators were quantified as 0.56 Hz/kPa (resonator I) and −1.23 Hz/kPa (resonator II) in the pressure range from 200 kPa to 7000 kPa under a reference temperature of 20 °C. In addition, the temperature sensitivities were quantified as 1.95 Hz/°C (resonator I) and 1.83 Hz/°C (resonator II) at 0 °C under a reference pressure of 100 kPa.

## 3. Fabrication

**Figure 5:**
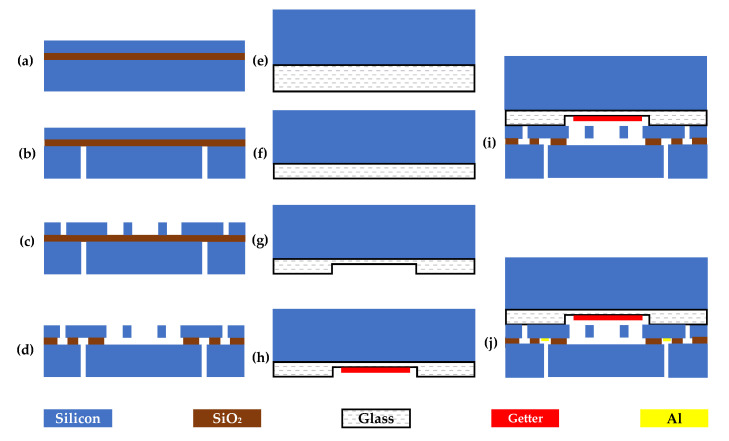
The fabrication processes of the sensor based on SOI-MEMS technologies. (**a**) Cleaning the SOI wafer; (**b**) conducting lithography to make through silicon vias on the handle layer; (**c**) conducting the second deep reactive ion etching (DRIE) on the device layer to form pressure-sensitive structures; (**d**) releasing resonant beams by HF; (**e**) conducting anodic bonding between a silicon wafer and a BF33 glass wafer; (**f**) thinning the glass layer of the SOG wafer by chemical mechanical planarization (CMP); (**g**) dry etching the glass wafer; (**h**) evaporating getters on the cavity; (**i**) conducting anodic bonding between the SOI and the SOG wafers; (**j**) evaporating aluminum electrodes for wire connections.

Simple SOI-MEMS processing technologies were used to fabricate the proposed resonant beams and through silicon vias (see Figure 5). In the fabrication process, a 4” SOI wafer (device layer: 40 µm, <100> oriented, p-type, doping concentration of 7.7 × 10^19^; oxide layer: 2 µm; and handle layer: 300 µm, <100> oriented, p-type, doping concentration of 6.6 × 10^14^~13 × 10^14^), a 4” BF33 glass wafer with a thickness of 500 μm and a 4” silicon wafer with a thickness of 1500 μm were employed in device fabrication. The main fabrication steps included deep reactive ion etching, hydrofluoric (HF) releasing and anodic bonding.

First, the SOI wafer was immersed in deionized water and dried with pure N_2_ gas after being cleaned by piranha etchant to remove organic residues and boiled deionized water to remove soluble ions (see Figure 5a). Second, 100 nm Cr was sputtered on the device layer to protect the device layer during the etching step on the handle layer, and then, using patterned photoresist as a mask, the handle layer of the SOI wafer was etched through to the oxide layer, forming the through silicon vias (see Figure 5b). Third, employing patterned photoresist and the Cr film as a compound mask, the device layer of the SOI wafer was etched through to the oxide layer, forming the sensitive structures (see Figure 5c). Fourth, lifting off the photoresist and Cr on the wafer was conducted based on the same process of the first step to clean the wafer. Additionally, the resonant beams were released by dry etching, employing HF solution and isopropanol in an alternate process (see Figure 5d).

The SOG wafer was made by conducting anodic bonding between a silicon wafer and a BF33 glass wafer (see Figure 5e). Additionally, the glass layer of the SOG wafer was thinned by chemical mechanical planarization (CMP) (see Figure 5f). The cavities for containing the vibration of the resonators in the BF33 glass wafer were drilled by HF dry etching (see Figure 5g). Then, a Ti/Au thin film was evaporated on the cavity as the getter material for gas absorption during the next anodic bonding process (see Figure 5h).

After finishing the fabrications of the SOI and the SOG wafers, anodic bonding was utilized to form a vacuum encapsulation for resonators where the voltage, vacuum level, tool pressure and temperature of anodic bonding were set at 400 V, 0.1 Pa, 100 kPa and 350 °C, respectively (see Figure 5i). The vacuum level of the vacuum cavity of the microsensor after anodic bonding was about 10~20 Pa. Then, aluminum films were evaporated on the TSVs of the bonding wafer to form electrical connections by a hard mask which was a glass wafer with through glass vias (TGVs) fabricated by laser processing (see Figure 5j). For isolating the silicon side walls, the diameter of TGVs (0.4 mm) on the glass wafer were smaller than TSVs (0.6 mm) on the handle layer of the SOI wafer. Ohmic contacts were formed by annealing the wafer in a 450 °C furnace for 30 min.

**Figure 6:**
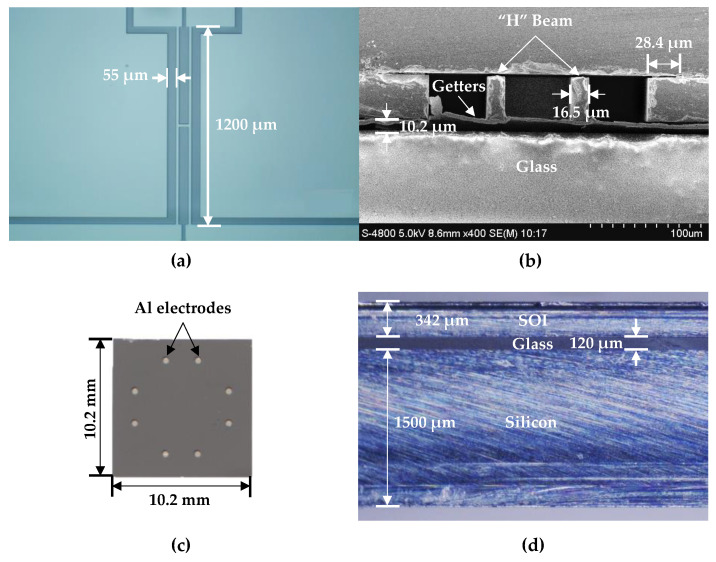
The fabrication results: (**a**) top view of the “H” resonant beam; (**b**) an SEM cross-section image of the microsensor; (**c**) top view of the microsensor and (**d**) side view of the microsensor.

Figure 6 shows the fabrication results of the microsensor, containing the top view of the resonator (see Figure 6a), the scanning electron microscopy (SEM) cross-section image of the resonator (see Figure 6b), the top view of the microsensors after dicing (see Figure 6c) and the side view of the microsensor (see Figure 6d).

**Figure 7:**
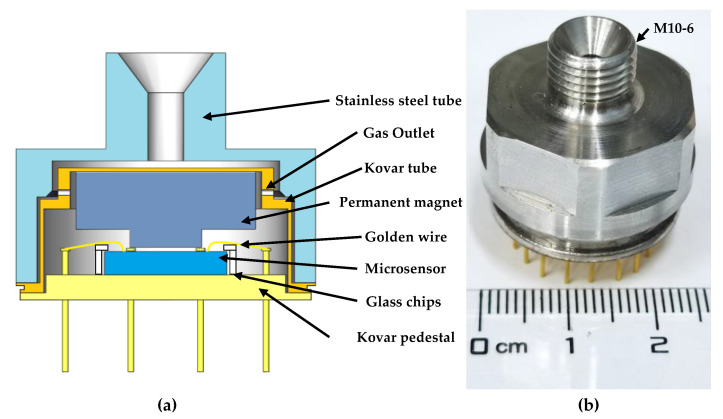
(**a**) The cross-section image of the resonant pressure sensor’s packaging concept; (**b**) an image of the packaged microsensor.

As shown in Figure 7a, the fabricated microsensor was fixed to the Kovar pedestal by glass chips. Electrodes on the microsensor and the pins of the Kovar pedestal were connected by ball soldering based on golden wires. One permanent magnet was fixed on the Kovar tube to produce a magnetic field of ~1 T in the package. The stainless steel tube, Kovar tube and Kovar pedestal were connected by double laser welding to maintain a high strength. Moreover, gas outlets in the Kovar tube were used to form gas paths during measurements. The package prototype is shown in Figure 7b.

## 4. Characterization

The open-loop characterization was used to obtain some properties of the resonant pressure senor, such as quality factor, intrinsic frequency, signal strength and phase drift of the two resonators. First, the sensor was fixed on the open-loop circuit, which can reinforce the output signal and was connected with a network analyzer. The network analyzer was used to supply an analog signal to one side of the “H” beam and pick up the signal induced by the other side of the “H” beam.

**Figure 8:**
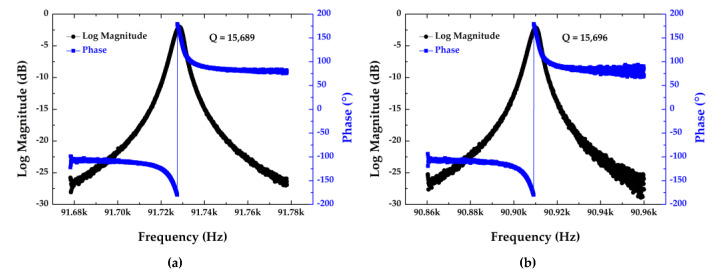
The properties of (**a**) resonator I and (**b**) resonator II under an atmospheric pressure of 100 kPa and a room temperature of 25 °C.

In this way, the resonant frequency of resonator I was quantified as 91.729 kHz with the phase drift of ~160.2° under a quality factor of 15,689 (see Figure 8a). Meanwhile, the resonant frequency of resonator II was quantified as 90.910 kHz with a phase drift of ~162.7° under a quality factor of 15,696 (see Figure 8b).

In order to further characterize the performances of the fabricated sensor, a closed-loop circuit producing self-oscillation signals was developed. The voltage generated by the vibration of the resonator was amplified by an amplifier, and then the voltage was lowered and sent to the driving beam of the resonator for excitation in a closed loop. In order to maintain the stable vibrations of the resonator, an AGC module, including a bandpass filter, a comparator and a field effect transistor, was introduced into the closed-loop circuit.

A pressure controller (PPC4, FLUCK) and a temperature chamber (SU-241, ESPEC) were employed to provide pressure measurements and surrounding temperatures during the characterization processes. In this study, the sensor was characterized within a pressure range from 200 kPa to 7000 kPa and a temperature range from −40 °C to 80 °C.

**Figure 9:**
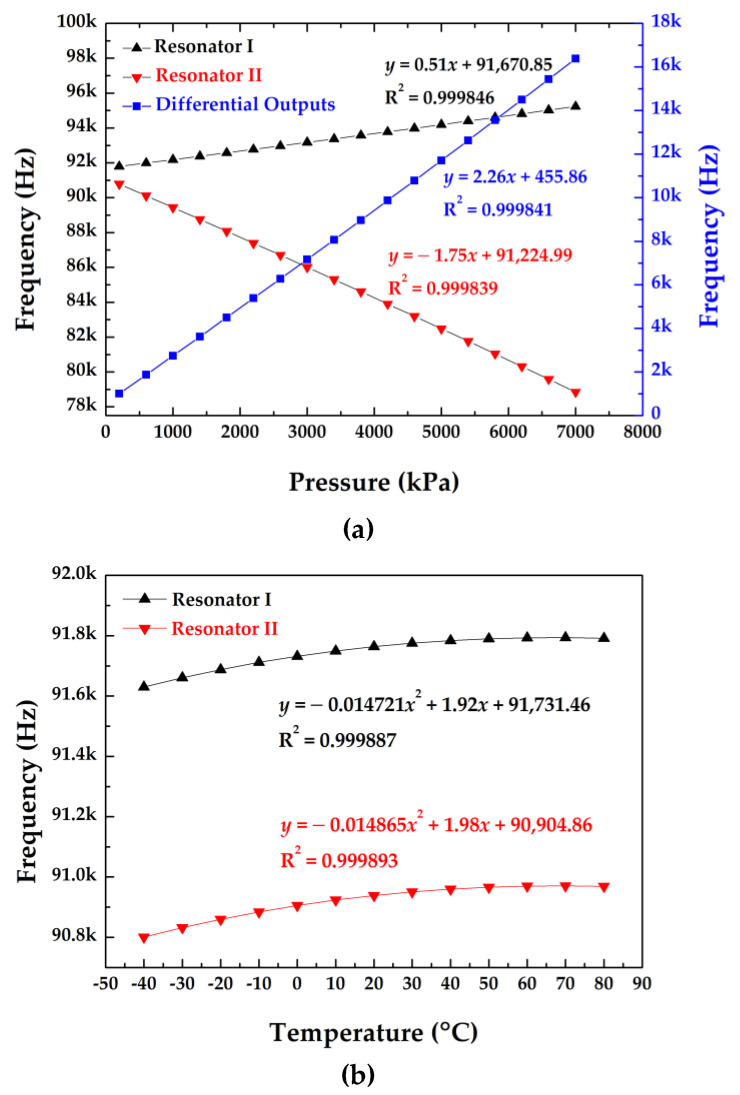
(**a**) The pressure–frequency curve of the developed sensor at 20 °C; (**b**) the temperature–frequency curve of the developed sensor under atmosphere pressure of ~100 kPa.

Figure 9a shows the intrinsic frequencies of two resonators as a function of pressure at 20 °C, producing the sensitivities and linearly dependent coefficients of 0.51 Hz/kPa and 0.999846 for resonator I; −1.75 Hz/kPa and 0.999839 for resonator II, which were consistent with the simulation results. Figure 9b shows the intrinsic frequencies of the two resonators as a function of temperature. The temperature sensitivity of the differential outputs was quantified as 0.06 Hz/°C in the temperature range from −40 to 80 °C under an atmosphere pressure of ~100 kPa. This value cannot match the result of the simulation due to the machining errors during the fabrication process.

**Figure 10:**
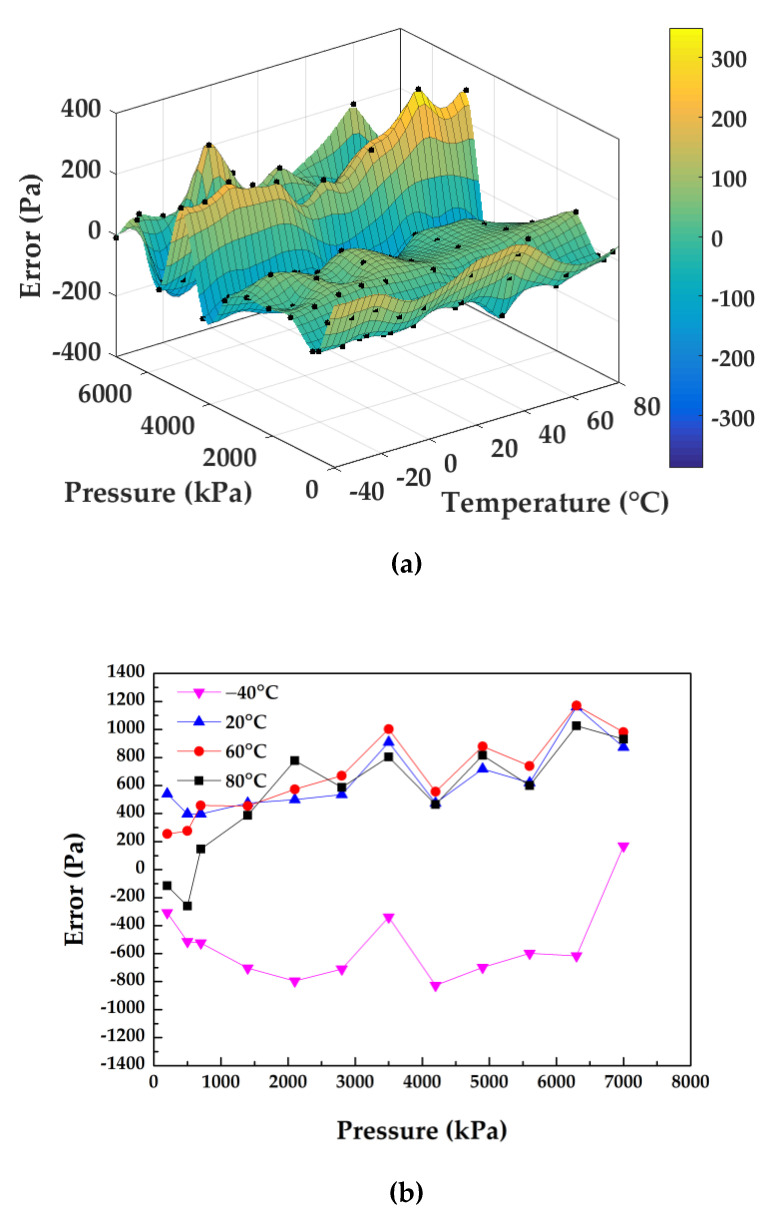
(**a**) The surface fitting of the sensor from the calibration process; (**b**) the measurement errors curve at −40 °C, 20 °C, 60 °C and 80 °C in three cycles.

Moreover, calibrations were conducted to verify the high performance of the microsensor. Figure 10a shows the fitting errors of the fabricated sensor in the full pressure and temperature ranges with temperature compensation based on differential outputs and a temperature sensor, producing compensation errors within ±400 Pa with corresponding ±0.006% FS, which indicated that the developed sensor was stable enough under the heat conditions from −40 °C to 80 °C. Figure 10b shows the measurement errors of the sensor at the surrounding temperatures of −40 °C, 20 °C, 60 °C and 80 °C in three cycles, which demonstrated a high accuracy with quantified maximum measurement errors within ±1170 Pa and a corresponding ±0.017% FS.

## 5. Conclusions

A design of the resonant pressure sensor with a wide pressure measurement range was presented, where silicon islands were used to increase equivalent stiffness of the pressure-sensitive diaphragm; the SOG cap was employed to decrease the temperature sensitivity; and the cylindrical vacuum cavity was used to increase the strength of the microsensor. Meanwhile, FEA simulations were used to calculate the pressure sensitivities and temperature sensitivities. According to the simulations, the sensitivities of the two resonators were 0.56 Hz/kPa and −1.23 Hz/kPa, with a differential linearity of 0.999939. The microsensor was fabricated with MEMS techniques and simple processing technologies only including two DRIE steps. The experimental results showed that the Q-factors of the resonator were quantified to be higher than 15,000, which showed that the two resonant beams of the microsensor worked in a high vacuum. Besides, the pressure sensitivities of the two resonators were 0.51 Hz/kPa and −1.75 Hz/kPa, with a differential linearity of 0.999841. Additionally, the temperature coefficient of the differential frequency was 0.06 Hz/°C, which means that the frequency drift caused by temperature was less than 8 Hz from −40 °C to 80 °C. More in-depth characterizations based on the closed-loop self-oscillation system showed that the prototype demonstrated low fitting errors within 0.006% FS and low measurement errors within 0.017% FS under the pressure range from 200 kPa to 7000 kPa in the temperature range of −40 °C to 80 °C in three cycles.

## Figures and Tables

**Table 1:** Shape parameters of pressure-sensitive diaphragm [35].

Items	α
Square	0.31
Circle	0.24

**Table 2:** Parameters of chips with different vacuum cavity sizes.

Type	Length/mm	Width/mm	Diameter/mm
A	5	5	-
B	3.6	3.6	-
C	-	-	5
D	-	-	3.6

**Table 3:** Experimental results of pressure loading on chips with different sizes.

Type	11 MPa	20 MPa	35 MPa	40 MPa	60 MPa
A	Broken	-	-	-	-
B	OK	OK	Broken	-	-
C	OK	Broken	-	-	-
D	OK	OK	OK	OK	Broken

**Table 4:** Dimensional information of the microsensor model in FEA simulations.

Part	Length/μm	Width/μm	Thickness/μm	Depth/μm	Diameter/μm
Resonant Beam	1200	16.5	40	-	-
Device Layer of SOI	10,200	10,200	40	-	-
Handle Layer of SOI	10,200	10,200	300	-	-
Oxide Layer of SOI	10,200	10,200	2	-	-
Vacuum Cavity	-	-	-	10	3560
Glass Layer of SOG	10,200	10,200	120	-	-
Silicon Layer of SOG	10,200	10,200	1500	-	-

**Table 5:** Material properties in FEA simulation.

Item	Silicon	BF33
Young’s modulus (GPa)	165	64
Density ($g/{\mathrm{cm}}^{3}$)	2.33	2.23
Poisson’s ratio	0.28	0.2
